# Preventing Suicidal Thoughts and Behaviors Among Youth: Integrative Data Analysis of Crossover Impacts of the Coping Power Preventive Intervention

**DOI:** 10.1016/j.jaacop.2025.01.005

**Published:** 2025-02-07

**Authors:** Heather L. McDaniel, Alexa C. Budavari, Alexandra Tonigan, Ava E. Michael, Stephen G. West, Nisha Gottfredson O’Shea, Nicole P. Powell, Lixin Qu, Lissette M. Saavedra, Anna Yaros, Catherine P. Bradshaw, John E. Lochman, Antonio A. Morgan-López

**Affiliations:** a University of South Carolina, Columbia, South Carolina; b University of Virginia, Charlottesville, Virginia; c RTI International, Research Triangle Park, North Carolina; d Arizona State University, Tempe, Arizona; e Free University of Berlin, Berlin, Germany; f University of Alabama, Tuscaloosa, Alabama

**Keywords:** evidence-based interventions, integrative data analysis, mindfulness, prevention, suicidality

## Abstract

**Objective:**

Despite evidence-based interventions for psychiatric disorders that often precede suicidality, suicide remains a leading cause of death among youth. There has been increased interest in whether preventive interventions targeting early risk factors lead to decreased distal risk for suicidal thoughts and behaviors (STBs). This study examined the impact of Coping Power (CP), a school-based preventive intervention targeting externalizing problems, on STBs.

**Method:**

The sample included 3,182 youths (36.4% female; 77.3% Black) who participated in 1 of 11 randomized controlled trials of CP. Individual-level data across trials were harmonized using integrative data analysis to address cross-study variation in measurement of STBs. The study used meta-analysis of individual participant data for modeling cross-study variation in intervention effects and propensity score weighting for addressing covariate imbalance arising from combining intervention arms across studies. Hypothesis tests were conducted for parent- and teacher-reported STBs under propensity score–weighted multilevel modeling.

**Results:**

Compared with school as usual, youth participating in mindfulness-enhanced CP demonstrated significant decreases in parent-reported STBs over time (*b* = −.08 [.02], *p* < .001; after 1 year: *d* = −0.13; after 2 years: *d* = −0.25), and youth participating in Internet-enhanced CP demonstrated significant decreases in teacher-reported STBs over time (*b* = −.08 [.03], *p* = .003; after 1 year: *d* = −0.20; after 2 years: *d* = −0.40). Inconsistent results for standard CP and individual CP in sensitivity analyses preclude clear conclusions for these 2 intervention formats.

**Conclusion:**

Synthesis of the reported findings highlights the promise of digital health and mindfulness-based interventions for youth with externalizing problems in reducing STBs. Additional research is needed to better understand the nature of for whom, how, and under what conditions preventive interventions impact later STBs.

**Diversity & Inclusion Statement:**

We worked to ensure sex and gender balance in the recruitment of human participants. We worked to ensure race, ethnic, and/or other types of diversity in the recruitment of human participants. We worked to ensure that the study questionnaires were prepared in an inclusive way. One or more of the authors of this paper self-identifies as a member of one or more historically underrepresented racial and/or ethnic groups in science. One or more of the authors of this paper self-identifies as a member of one or more historically underrepresented sexual and/or gender groups in science. One or more of the authors of this paper self-identifies as living with a disability. One or more of the authors of this paper received support from a program designed to increase minority representation in science. We actively worked to promote sex and gender balance in our author group. We actively worked to promote inclusion of historically underrepresented racial and/or ethnic groups in science in our author group. The author list of this paper includes contributors from the location and/or community where the research was conducted who participated in the data collection, design, analysis, and/or interpretation of the work. While citing references scientifically relevant for this work, we also actively worked to promote inclusion of historically underrepresented racial and/or ethnic groups in science in our reference list. While citing references scientifically relevant for this work, we also actively worked to promote sex and gender balance in our reference list.

## Plain language summary

There has been increased interest in preventive interventions that target risk factors for suicidal thoughts and behaviors. In this study, we analyzed data from 3,182 youth (36.4% female youth; 77.3% Black youth) who participated in one of 11 randomized controlled trials of the Coping Power program, a school-based preventive intervention for youth demonstrating externalizing problems. Youth participating in versions of Coping Power that were optimized with online content or mindfulness-based content had reduced suicidal thoughts and behaviors, as compared to youth participating in school as usual. Our findings highlight the promise of digital health and mindfulness-based interventions in reducing suicidal thoughts and behaviors among youth with externalizing problems.

Suicide is the second leading cause of death among youth ages 10 to 19 in the United States.^1^ Among high school students, almost 1 in 5 reported thinking about suicide, and about 1 in 10 reported having a plan to die by suicide.^2^ Despite evidence-based behavioral and pharmacological treatments for psychiatric disorders that are often precursor conditions to suicidality (eg, depression, anxiety), rates of suicidal thoughts and behaviors (STBs) have remained stable for male youth and have increased for female youth and certain subgroups of male youth.^3^ Further, death by suicide has increased for some demographic groups, such as Black youth, over the last decade.^4^

Because it has been difficult to reduce suicide rates by treating psychiatric disorders as they emerge during late adolescence and young adulthood, there has been increased interest in the impact of early preventive interventions on distal suicide risk. Such efforts have typically focused on early programming in childhood and adolescence,^5^ including crossover effects of preventive interventions, that is, impacts on STBs where STBs were not the original target of intervention.^6^^,^^7^ A small number of preventive interventions implemented in childhood that did not target suicidality explicitly (eg, Familias Unidas,^8^ Family Check-up,^9^ Family Bereavement Program^10^) have shown effects on reductions in suicide attempts and other self-harm behaviors 5 to 15 years after the intervention. However, gaps in this literature remain. As Brent^11^ noted, these interventions primarily target internalizing symptoms; yet interventions focused on the externalizing pathways to suicidality have been inadequately studied with regard to distal effects of STBs. This is critical because there may be various pathways to suicidality. For example, whereas female adolescents who die by suicide are more likely to have a previous internalizing disorder diagnosis, male adolescents who die by suicide are more likely to have had an externalizing problem or attention-deficit/hyperactivity disorder diagnosis.^4^^,^^12^ Many programs focused on externalizing problems target skill sets that are also addressed in programs focused on internalizing problems (eg, emotion regulation, adaptive cognitions, social skills) and comprise core components that have transdiagnostic utility (eg, cognitive restructuring, behavioral coping strategies).^13^ Identifying the potential benefits of these interventions beyond specific externalizing outcomes, or so-called crossover effects,^7^ will help to demonstrate the utility of these programs for youth facing a variety of mental health challenges, with potential implications for long-term suicide prevention.

### Potential Crossover Effects of the Coping Power Program on STBs

Coping Power (CP) is one such program that seems likely to generate crossover effects on STBs. This school-based preventive intervention was originally designed to reduce externalizing symptoms for youth already demonstrating elevated levels of behavioral problems.^14^ The program leverages 2 of the most frequently used approaches, cognitive and behavioral,^15^ to support students demonstrating elevated aggressive behavior in schools. CP typically includes youth- and parent-group components that are delivered by trained clinicians. CP is theoretically based in the contextual social-cognitive model of aggression, targeting improvement in youth emotion regulation, social-cognitive appraisals, and social problem solving in the youth-focused group sessions as well as improving consistency in parenting strategies via the parent-focused group curriculum.^14^

Prior randomized controlled trials (RCTs) of CP have shown effects on externalizing behaviors, including substance use, aggression, and delinquency, while promoting social competence and concentration.^14^ Additionally, prior research suggests that CP may reduce broadband internalizing problems among youth^13^^,^^14^; however, the effect of CP on STBs remains unexplored. Moreover, there has been less systematic examination of differential effects of different versions of CP that have been developed over the last 20 years (see review by McDaniel *et al.*^16^). Specifically, some versions of CP have been tested that included particular enhancements, such as mindfulness components, which may be particularly beneficial for addressing STBs.^17^^,^^18^ However, to date, CP has not been examined for crossover effects on STBs.^19^

### Current Study

One difficulty in estimating intervention effects on STBs within any single RCT is the fact that STBs are low base rate outcomes that can compromise the power to detect intervention effects.^8^^,^^11^^,^^20^ To address this challenge, Reider and Sims called for “pool[ing] data across prevention studies in order to increase the statistical power available to examine the impact of prevention interventions on [STBs]”^7^^(pS6)^ under the integrative data analysis (IDA) framework (see also Ayer *et al.*,^6^ Brent,^11^ and Pearson and Sims^21^) ^22^^,^^23^ IDA is a cross-study framework for estimating commensurate scale scores for a construct when pooling individual-level data across studies; one of the long-noted major advantages of pooling datasets under IDA has been the increase in power for low base rate outcomes.^24^

In the current study, we leveraged IDA to examine the crossover effects of CP on STBs using data from 11 CP trials, with particular interest in the impact of different variations of CP. Given that CP contains components that may have promise for transdiagnostic impacts on other outcomes,^13^ we hypothesized that all forms of CP would demonstrate long-term reductions in suicidality compared with a school-as-usual (SAU) control group. Additionally, we hypothesized that the mindfulness-enhanced version of CP in particular would demonstrate impacts on youth suicidality^17^^,^^18^ because of its enhanced focus on self-regulation, attentional capacity, parental warmth, and positive discipline.^25^ Moreover, preliminary evidence suggests that mindfulness-based interventions demonstrate clinically meaningful reductions in STBs.^26^^,^^27^ To examine these research questions, our study approach leveraged IDA to address cross-study variability in the measurement of STBs,^24^^,^^28^ meta-analysis of individual patient data for characterizing cross-study variability in the intervention effects of interest,^29^ and propensity score weighting for addressing covariate imbalance that can occur when combining treatment arms across RCTs.^19^^,^^30^

## Method

### Participants

The sample for the present study included 3,182 youths who participated in 1 of 11 RCTs of CP (see study protocol^19^) conducted in schools across 3 southern and southeastern states. Participating youth were in fourth to ninth grade at baseline and screened into the respective trial based on teacher- and sometimes parent-screener scores indicating elevated levels of aggressive behavior. Of participating youth across the 11 RCTs, 36.4% were female, and 77.3% were Black. Additional demographic information is reported in Table 1.

**Table 1:** Sample Descriptive Statistics at Baseline

Variable	SAU (no. studies = 9; no. youth = 1,164)		SCP (no. studies = 10; no. youth = 1,460)		ICP (no. studies = 2; (no. youth = 210)		MCP (no. studies = 1; no. youth = 52)		IECP (no. studies = 1; no. youth = 49)		Active control (no. studies = 2; no. youth = 247)		Pre-weight *p*	Post-weight *p*
	Mean	(SD)	Mean	(SD)	Mean	(SD)	Mean	(SD)	Mean	(SD)	Mean	(SD)		
Grade level	6.03	(2.03)	5.72	(1.96)	4	(0)	4	(0)	4	(0)	4	(0)	.02	.63
	**%**		**%**		**%**		**%**		**%**		**%**			
Female youth	37.8		35.5		37.1		36.5		40.8		36.4		.87	.95
Black youth	76.8		77.0		72.4		90.4		89.8		86.6		<.001	.94
Family income													.08	.55
None	5.4		6.1		5.2		9.8		4.1		4.5			
<$15,000	28.3		28.3		24.8		23.5		32.7		40.7			
$15,000-$29,999	28.9		26.8		30.5		25.5		30.6		22.8			
$30,000-$49,999	24.2		25.5		26.2		33.3		16.3		18.7			
>$50,000	13.2		13.4		13.3		7.8		16.3		13.4			
	**Mean**	**(SD)**	**Mean**	**(SD)**	**Mean**	**(SD)**	**Mean**	**(SD)**	**Mean**	**(SD)**	**Mean**	**(SD)**		
Parent-reported STBs score	−0.03	(0.65)	−0.03	(0.65)	0.01	(0.68)	−0.06	(0.67)	−0.17	(0.46)	−0.12	(0.56)	.15	.22
Teacher-reported STBs score	−0.16	(0.40)	−0.16	(0.39)	−0.16	(0.42)	−0.27	(0.19)	−0.23	(0.39)	−0.20	(0.44)	.22	.63

Note: Multilevel model *p* values are based on propensity score–weighted multilevel models that account for study-level fixed effects and school-level random effects. ICP = individual Coping Power; IECP = internet-enhanced Coping Power; MCP = mindfulness-enhanced Coping Power; SAU = school as usual; SCP = standard Coping Power; STBs = suicidal thoughts and behaviors.

### Procedure

Data from the 11 RCTs of CP were harmonized under the IDA framework in which individual-level data from the original trials were combined and analyzed as a single dataset.^31^ Parent-reported STBs were assessed across all 11 studies, with 2 to 8 waves of data collection per study, which covered approximately 1 to 7 years of follow-up. Teacher-reported STBs were assessed in 10 of 11 studies, with 3 to 7 waves of data collection per study, which also covered approximately 1 to 7 years of follow-up. Tables S1 and S2, available online, provide more information about STBs data collected by study and wave.

#### Intervention Conditions

Several versions of CP were delivered across the 11 RCTs included in this article. In each trial, at least 1 treatment arm included the implementation of CP. Ten of the studies included randomization to the originally developed CP (ie, standard CP), as described in “Potential Crossover Effects of the Coping Power Program on STBs” (see also Lochman *et al.*^14^). Two of the trials included randomization to individual CP, which contained the same content as the youth-focused CP groups but was delivered one-on-one. Two trials included optimized versions of CP that integrated online modules (ie, Internet-enhanced CP) or mindfulness content (ie, mindfulness-enhanced CP). Two of the trials included randomization to an active control condition, and 9 of the trials included an SAU comparison condition. Each intervention condition (ie, standard, individual, Internet-enhanced, and mindfulness-enhanced CP) and the active control condition were compared with the SAU condition in our analyses. For additional details on the 11 RCTs, see Morgan-López *et al.*^19^

### Measures

#### Demographic Variables

Demographic predictors included grade in school, sex, race, and family income. Differences across intervention conditions (unweighted and propensity score weighted) are shown in Table 1.

#### Suicidal Thoughts and Behaviors

In 9 of the RCTs, parents and teachers completed the Behavior Assessment System for Children (BASC/BASC-2).^32^^,^^33^ In 2 of the RCTs, parents and teachers completed the Achenbach System of Empirically Based Assessment (ASEBA).^34^ Across both assessment systems, 5 items (asked of both parents and teachers) were available that had content related to STBs: 3 items from the BASC (Tries to hurt self; Says “I want to die” or “I wish I were dead”; Says, “I want to kill myself”) and 2 items from the ASEBA (Talks about killing themselves; Deliberately harms self or attempts suicide). These 5 items were evaluated for whether any of the items could be harmonized across assessment systems (ie, similar item content to warrant a combined BASC/ASEBA item) using expert rating procedures for semantic harmonization of transdiagnostic item content developed in McDaniel *et al.*^35^ Of the 5-item pool, only the BASC item “Says, ‘I want to kill myself’” and the ASEBA item “Talks about killing themselves” were judged as harmonizable. The remaining BASC items were treated as missing in studies with the ASEBA assessment and vice versa; this yielded a 4-item construct, but with some item-level missingness at the study level on all items except the harmonized “Says, ‘I want to kill myself’/Talks about killing themselves” item, which is common in IDA studies and can be addressed with modern missing data approaches to scale score estimation (eg, see Bauer and Hussong^28^). Item responses for all items were recoded to binary (ie, 0 = “Never/Not True”; 1 = all other responses) because of insufficient variability in the higher STBs item categories. Parent- and teacher-reported STBs were modeled separately, as described in greater detail in the following sections.

### Analysis Plan

#### Zero-Inflated Mixture—Moderated Nonlinear Factor Analyses

An initial nonlinear factor analysis was conducted in M*plus* version 8^36^ to ensure unidimensionality of the harmonized measures of teacher- and parent-reported STBs. We used weighted least squares, mean, and variance adjusted to account for the ordered categorical nature of the items and cluster-robust standard errors to account for nesting of youth within schools.^37^ Model fit was assessed using standard fit indices suggested to indicate adequate fit of the model to the data (ie, comparative fit index ≥ 0.95, root mean square error of approximation ≤ 0.05).^38^

We then fit moderated nonlinear factor analysis (MNLFA) models to assess differential item functioning (DIF).^28^^,^^39^ Given that STBs are relatively rare (ie, many participants will be asymptomatic, resulting in an excess of zeroes in the data), a zero-inflated mixture (ZIM) MNLFA was fit to estimate 2 factor scores^40^: parent-reported suicidality and teacher-reported suicidality. Intercept DIF is present if an item differs in prevalence for some groups, controlling for underlying STBs; factor loading DIF is not estimated in ZIM MNLFA.^40^ Intercept DIF was explored across grade in school, gender, race, family income, and study wave; DIF could not be assessed by measure because only a single item was common to the BASC and ASEBA. We estimated and output scale scores that accounted for DIF, if present. Modeling was conducted separately for each reporter. The scale of the factor was set to N(0,1) at baseline. If a DIF parameter was statistically significant at the *p* < .001 level, the parameter was retained and included in the final scoring model. The use of α = .001 is based on a Bonferroni correction to adjust for multiple testing across DIF comparisons. After fitting the final ZIM MNLFA model, items were scored, and scale scores were estimated for all youth based solely on the item parameters from the symptomatic class as per Saavedra *et al.*^40^ This procedure ensures that youth in the symptomatic and asymptomatic classes had factor scores on the same scale that was derived from the symptomatic youth. Factor scores then served as the outcome variables in subsequent analyses.

Across the full sample, 22% of cases were missing parent-reported STBs across all waves (Table S1, available online, contains additional information including the percentage of data collected by study and wave). Alternatively, 13.1% of cases were missing teacher-reported STBs across all waves (Table S2, available online, includes the percentage of data collected by study and wave). Cases with completely missing data across time did not contribute information to the subsequent outcome analyses.

#### Propensity Score Weighting

Because intervention conditions were combined across the 11 RCTs of CP in a manner that could potentially compromise covariate balance achieved through randomization within each original trial, we estimated multilevel propensity score weights.^41^ We used PROC GLIMMIX (SAS Institute Inc, Cary, NC) to balance covariates across treatment conditions (ie, standard CP, individual CP, Internet-enhanced CP, mindfulness-enhanced CP, active control condition) and the SAU control condition. The use of these weights in outcomes analysis produces unbiased estimates of focal intervention effects in the combined IDA sample. The covariates listed under “Measures” were examined for their joint relations to both treatment assignment and reported STBs for inclusion in propensity weight models as recommended by Steiner *et al.*^42^ After this selection process, covariates were included in multilevel multinomial logistic regression models to estimate probabilities of treatment assignment. Probabilities of treatment assignment were converted to weights by taking their inverses. Following weighting, standardized mean differences at baseline did not exceed *d* = |0.10| and were not statistically significant, mimicking covariate balance of a multiarm, multisite randomized experiment. Propensity weights were estimated separately for parent- and teacher-reported STBs.

#### Hypothesis Tests

Hypothesis tests were conducted separately for parent- and teacher-reported STBs under propensity score–weighted multilevel linear modeling using PROC HPMIXED (SAS Institute Inc) to account for 3 potential levels of clustering: repeated observations within participants, participants clustered within schools, and schools clustered within studies. Participant-level random effects for the intercept and slopes over continuous time in years were included in all models (Tables S1 and S2, available online, contain more information about the conversion of study waves to continuous time in years). Model estimates were then converted into model-based longitudinal Cohen *d* effect sizes by using the methods of conversion outlined in Feingold,^43^ using a pooled SD at baseline across all conditions. As such, we present Cohen *d* at 1 year and 2 years after the study started, reflecting approximately postintervention assessment and 1-year postintervention across studies. Missing data were addressed using restricted maximum likelihood, which assumes that data are missing at random conditional on the observed variables in the model.^44^

## Results

### Zero-Inflated Mixture—Moderated Nonlinear Factor Analyses

Initial ordinal item confirmatory factor analyses showed evidence of unidimensionality for both the parent-reported (comparative fit index = 0.99, root mean square error of approximation = 0.041) and teacher-reported (comparative fit index = 0.99, root mean square error of approximation = 0.035) measures of STBs. Results of the ZIM MNLFA analyses suggested that there was not statistically significant DIF on parent- or teacher-reported STBs related to predictors (ie, grade, age, gender, race, family income, and study wave) at the *p* < .001 level. As such, the final ZIM MNLFA scoring parameters (Table 2) did not include DIF parameters. Item parameter estimates for the symptomatic STBs class (Table 2) were applied to the full sample (ie, symptomatic and asymptomatic cases) per Saavedra *et al.*,^40^ and factor scores were extracted for use as outcomes in the subsequent outcome analyses.

**Table 2:** Assessment of Suicidal Thoughts and Behaviors (STBs): Items, Endorsement, Loadings, and Thresholds

Construct/Items	Percentage Endorsing	Factor Loading	Threshold
Parent-Reported STBs			
Tries to hurt self (BASC)	3.7%	1.21	2.48
Says “I want to die” (BASC)	15.8%	4.07	3.60
Says “I want to kill myself”/talks killing self (harmonized)	6.6%	2.12	2.80
Deliberately harms self/attempts suicide (ASEBA)	0.7%	0.89	2.69
Teacher-reported STBs			
Tries to hurt self (BASC)	5.3%	2.55	2.54
Says “I want to die” (BASC)	3.3%	1.86	2.31
Says “I want to kill myself”/talks killing self (harmonized)	2.8%	1.49	2.31
Deliberately harms self/attempts suicide (ASEBA)	3.9%	0.88	2.10

Note: Parameters for the symptomatic class are shown, but were used to score all observations.^40^ In the symptomatic class, the above factor loading and threshold estimates were significant at *p* ≤ .01. ASEBA = Achenbach System of Empirically Based Assessment; BASC = Behavior Assessment System for Children.

### Estimating Propensity Score Weights

Propensity score weights were estimated for use in subsequent outcomes analyses. Each model generating the propensity score weights included different covariates across outcomes and study inclusion (all participants, fourth graders only). Covariate balance was tested via multilevel multinomial logit models for outcome model–specific propensity score weights and are reported in the following sections.

#### Parent-Reported STBs: All 11 Studies

The final propensity weight model included study-level means for baseline parent-reported STBs (*F*_5,3165_ = 50.35, *p* < .001) and grade level (*F*_5,3165_ = 40.62, *p* < .001). Covariate balance was not achieved for individual CP due to limited grade variability (all fourth graders) and differences in baseline parent-reported STBs between individual CP and SAU (Table 1); that is, there were too few fourth graders in SAU who also had comparable levels of baseline parent-reported STBs as fourth graders in individual CP. This issue necessitated a sensitivity analysis that restricted propensity weight/outcome models to fourth graders.

#### Parent-Reported STBs: Fourth Graders Only

The final propensity weight model included individual-level baseline scores of parent-reported STBs (*F*_5,1779_ = 2.48, *p* = .03) and race (*F*_5,1779_ = 5.28, *p* < .001).

#### Teacher-Reported STBs: All 11 Studies

The final propensity weight model included school-level means for baseline teacher-reported STBs (*F*_5,3171_ = 11.61, *p* < .001).

#### Teacher-Reported STBs: Fourth Graders Only

The final propensity weight model included only race (*F*_5,1789_ = 5.39, *p* < .001).

### Hypothesis Testing

Each outcome model included the outcome-specific propensity score weights that are discussed under “Estimating Propensity Score Weights.”

#### Parent-Reported STBs: All 11 Studies

Students in the SAU condition showed an increase in parent-reported STBs over time (*b* = .02 [.01], *z* = 1.96, *p* = .050). Compared with the SAU group, students participating in mindfulness-enhanced CP demonstrated statistically significant decreases in parent-reported STBs over time (*b* = −.08 [.02], *z* = −3.38, *p* < .001, with *d* = −0.13 at 1 year and increasing to *d* = −0.25, at 2 years). Students in individual CP showed significant decreases in parent-reported STBs over time compared with SAU (*b* = −.03 [.01], *z* = −2.68, *p* = .007, with *d* = −0.05 at 1 year and reaching *d* = −0.09 at 2 years). However, it is important to note that there was a failure to achieve covariate balance across SAU and individual CP conditions (*b* = .13 [.04], *z* = 3.00, *p* = .003) due to the lack of comparable cases on baseline parent-reported STBs in individual CP.

#### Parent-Reported STBs: Fourth Graders Only

Parent-reported STBs for students in the SAU condition showed no changes over time (*b* = .02 [.01], *z* = 1.33, *p* = .184). Compared with students in SAU, students in mindfulness-enhanced CP showed statistically significant decreases in parent-reported STBs over time (*b* = −.06 [.02], *z* = −2.40, *p* = .016, with *d* = −0.09 at 1 year and *d* = −0.19 at 2 years). In contrast to the full study sample, in the fourth-grade sample, there was not a statistically significant impact of individual CP on STBs over time (*b* = −.02 [.01], *z* = −1.74, *p* = .081), and in this subsample baseline balance was achieved (*b* = .08 [.04], *z* = 1.79, *p* = .073).

#### Teacher-Reported STBs: All 11 Studies

In contrast to parent-report, teacher-reported STBs for students in the SAU condition showed significant decreases across time (*b* = −.09 [.01], *z* = −7.34, *p* < .001). Compared with SAU, internet-enhanced CP was associated with statistically significant decreases in STBs reported by teachers over time (*b* = −.08 [.03], *z* = −2.94, *p* = .003, with *d* = −0.20 at 1 year and decreasing to *d* = −0.40 at 2 years). However, compared with SAU, standard CP was associated with a statistically significant attenuation of the decrease in teacher-reported STBs over time (*b* = .03 [.02], *z* = 2.19, *p* = .029, with *d* = 0.08 at 1 year and *d* = 0.15 at 2 years). Similarly, compared with SAU, individual CP was associated with a statistically significant attenuation of the decrease in teacher-reported STBs over time (*b* = .04 [.02], *z* = 2.28, *p* = .023, with *d* = 0.10 at 1 year and *d* = 0.20 at 2 years). These findings indicate that the overall decrease in STBs was more limited and suboptimal for individual CP and standard CP compared with SAU.

#### Teacher-Reported STBs: Fourth Graders Only

Teacher-reported STBs for students in the SAU condition showed significant decreases over time (*b* = −.05[.01], *z* = −4.37, *p* < .001). Compared with SAU, students participating in internet-enhanced CP showed statistically significant decreases in teacher-reported STBs over time (*b* = −.10 [.02], *z* = −4.67, *p* < .001, with *d* = −0.25 at 1 year and *d* = −0.50 at 2 years). Alternatively, individual CP was associated with a statistically significant attenuation of the decrease in teacher-reported STBs over time (*b* = .03 [.01], *z* = 2.64, *p* = .008 with *d* = 0.08 at 1 year and *d* = 0.15 at 2 years). However, in contrast to the full study sample, in the fourth-grade sample, there was not a statistically significant impact of standard CP on STB over time (*b* = .03 [.01], *z* = 1.92, *p* = .055).

## Discussion

There has been increased interest in the impact of early preventive interventions on later suicide risk, including crossover effects on STBs,^6^^,^^7^ where STBs were not the original target of intervention. Recent studies have begun to examine potential crossover effects of preventive interventions, yet this research has primarily focused on the impacts of interventions focused on internalizing problems. Less consideration has been given to potential crossover effects of interventions focused on externalizing problems in preventing STBs, but low base rates of STBs tend to contribute to single RCT examinations of STB outcomes being underpowered. The current study used an emerging individual-level data synthesis approach to the analysis of intervention crossover effects on STBs outcomes (see also Connell *et al.*^20^), leveraging IDA,^24^^,^^28^ meta-analysis of individual patient data,^29^ and propensity score weighting.^41^ We combined individual-level data for participants across 11 RCTs of CP to understand the longitudinal relation between different versions of CP and parent- and teacher-reported STBs, respectively.^14^^,^^19^

Results of analyses based on data from the 11 RCTs indicated that compared with students in the SAU condition, students in mindfulness-enhanced CP demonstrated statistically significant and meaningful decreases in parent-reported STBs that increased in magnitude over time. These findings align with research indicating that mindfulness-based therapies impact STBs.^26^^,^^27^ The mindfulness-enhanced CP program was designed based on theory and empirical evidence that mindfulness affects mechanisms in youth associated with reactive aggression (eg, attention, self-regulation) as well as parental warmth and parenting skills.^17^^,^^25^ These variables have in turn been linked to reduced risk for STBs, particularly for boys.^11^^,^^45^ Mindfulness-enhanced CP optimizes standard CP by integrating mindfulness strategies into both the youth and the parent components. This integration was achieved by adding focused sessions on mindfulness only to teach core mindfulness skills and infusing mindfulness into existing CP activities (eg, restructuring angry thoughts in CP vs acknowledging angry thoughts in mindfulness-enhanced CP). These components should augment CP by directly reducing STBs and reducing reactive aggression and peer rejection via additional cognitive restructuring.^25^^,^^46^ Additional research is needed to understand the exact pathways by which mindfulness-enhanced CP impacts STBs by specifying additional, preregistered mediation analyses.^19^

Our findings also indicated that compared with students in the SAU condition, Internet-enhanced CP demonstrated statistically significant and meaningful decreases in teacher-reported STBs over time. Internet-enhanced CP was created to be a briefer, more feasible hybrid program, with fewer youth and parent sessions implemented in school. Some of the original psychoeducational material (eg, anger management skills, steps of social problem solving) was moved online.^47^ Youth were highly engaged with the website, completing approximately 67% of activities on average. Lochman *et al.*^47^ reported that greater website use by children and parents was significantly related to lower teacher-reported conduct problems after assessment, controlling for baseline conduct problems. The online component included several enhancements that may have promoted both the initial outcomes reported by Lochman *et al.*^47^ and the reductions in STBs identified here. A chat function on the website allowed real-time support by the therapist; this feature may have enhanced the therapeutic alliance and, ultimately, the reductions in STBs. The online content may have also allowed youth to identify more with the psychoeducational content (eg, adaptable avatars). Because of increased individual engagement and decreased distraction from disruptive behaviors by peers in group sessions, children may have developed a more comprehensive understanding of CP mechanisms and skills (eg, emotion regulation).

Findings were mixed with regard to the impact of standard CP and individual CP on longitudinal STBs. Standard CP was associated with a statistically significant attenuation of the decrease in teacher-reported STBs over time in the full sample. However, in the fourth-grade sample, this effect fell to nonsignificance, precluding strong conclusions regarding the impact of standard CP on longitudinal STBs. Regarding individual CP, there was a similarly inconclusive pattern of results, and as noted in the results, balance was not achieved across individual CP and SAU in the full study sample. In the full sample analysis, students in individual CP showed significant decreases in parent-reported STBs over time compared with students in SAU. In contrast, in the fourth-grade sample, there was not a statistically significant impact of individual CP on parent-reported STBs over time. When examining teacher-reported STBs, individual CP was associated with a statistically significant suboptimal attenuation of the decrease in teacher-reported STBs over time in both the full sample and the fourth-grade sample. This consistent result of individual CP being associated with suboptimal teacher-reported outcomes warrants further investigation, inclusive of exploring for whom and under what conditions this occurs. However, no firm conclusions can be made about the effects of standard CP and individual CP on STBs.

This individual-level data synthesis approach had several strengths. Our approach leveraged IDA,^24^^,^^28^ meta-analysis of individual patient data,^29^ and propensity score weighting.^41^ Well-designed RCTs are typically touted as the gold standard design (see Cook^48^ for qualifications); however, a single RCT is very unlikely to have sufficient statistical power to detect crossover impacts on low-incidence outcomes such as STBs.^31^ Combining data from multiple RCTs using individual-level meta-analysis can provide sufficient data to achieve acceptable levels of statistical power. Analogous large multisite, multiarm RCTs carry a heavy implementation burden and incur escalating costs.^7^^,^^21^ In addition, data synthesis more adequately represents the heterogeneity present in multiple studies,^49^ facilitating broader generalization of the results. Our data synthesis approach also facilitated research questions and comparisons not planned in the original studies. For example, the intervention comparator in the mindfulness-enhanced CP pilot RCT was standard CP, which would be expected to lead to a greater decrease in STBs than SAU. Integration of the 11 datasets allowed a proper comparison between mindfulness-enhanced CP and SAU, conditions that did not overlap within this RCT originally.^30^ Further, our findings have significant implications for Black youth in particular (approximately 77% of the sample), as Black youth often have limited access to specialty mental health contexts; the promise of both school-based interventions with mindfulness components and delivery of CP online suggests a novel and untapped approach to prevention of STBs, even if STBs were not the original target of the intervention.

An important limitation of our approach is that our research questions undercut the within-study randomization structures of the original RCTs by combining intervention conditions across trials, creating a quasi-experiment in which potential imbalance among covariates could occur. To address this issue, propensity score weights were estimated and used to balance conditions successfully, with 1 exception. Covariate balance was not achieved for individual CP at a level resulting in a standardized mean difference at baseline of *d* < |0.10|. This result was likely due to limited grade variability (all fourth graders^19^). Given this imbalance and the inconsistent pattern of results, we do not believe we can make strong causal claims about the impact of individual CP on STBs. Similarly, there was an inconsistent pattern of impact of standard CP on teacher-reported STBs in the full study sample and fourth-grade sample. An additional important limitation is that propensity score weighting assumes that there are no unmeasured covariates that are related to both intervention arm assignment and the outcome variables.^50^ Further, no youth self-reports of STBs were available for this study.

In conclusion, our research leverages an innovative approach to synthesize and harmonize individual-level data across 11 RCTs with methodological variation.^19^ We focused on how an evidence-based externalizing behavior–focused preventive intervention, CP, affects STBs in youth over an extended period from childhood through adolescence. Our results suggested that optimized versions of CP, namely, internet-enhanced and mindfulness-enhanced CP, result in clinically meaningful reductions in STBs in youth. Preventive interventions such as CP that are widely implemented in schools provide broad access to critical prevention programming, particularly for youth who are less likely to receive specialty mental health care.^6^ Our findings highlight the promise of digital health and mindfulness-based interventions for youth with externalizing problems. Future analyses of these and other data are needed to better understand the nature of for whom, how, and under what conditions early preventive interventions impact later STBs.
