# Supplementary material for: Preventing Suicidal Thoughts and Behaviors Among Youth: Integrative Data Analysis of Crossover Impacts of the Coping Power Preventive Intervention
Source: JAACAP Open. 2025 Feb 7;3(3):467–76. doi: 10.1016/j.jaacop.2025.01.005 (PMC12414332; doi:10.1016/j.jaacop.2025.01.005)
Supplement: Supplemental Tables [file mmc1.pdf]

Table S1.

Percentage of Cases with Parent-reported Suicidal Thoughts and Behavior by Wave (Coded as Years from Baseline)

Study	*n*	Waves	Wave (coded as years from baseline)																		
			0	0.25	0.5	0.75	1	1.25	1.5	1.75	2	2.25	2.5	3	3.25	3.5	4	5	5.25	6.25	7.25
S1	241	4	100.00	0.00	0.00	0.00	91.29	0.00	0.00	0.00	85.06	0.00	0.00	79.25	0.00	0.00	0.00	0.00	0.00	0.00	0.00
S2	97	3	100.00	0.00	0.00	86.60	0.00	0.00	0.00	88.66	0.00	0.00	0.00	0.00	0.00	0.00	0.00	0.00	0.00	0.00	0.00
S3	245	6	100.00	0.00	0.00	0.00	90.61	0.00	0.00	0.00	87.76	0.00	0.00	82.86	0.00	0.00	79.59	85.71	0.00	0.00	0.00
S4	80	3	100.00	0.00	85.00	0.00	0.00	0.00	88.75	0.00	0.00	0.00	0.00	0.00	0.00	0.00	0.00	0.00	0.00	0.00	0.00
S5	360	8	100.00	98.06	0.00	0.00	0.00	90.56	0.00	0.00	0.00	85.83	0.00	0.00	79.17	0.00	0.00	0.00	78.61	79.72	81.94
S6	102	3	98.04	0.00	0.00	0.00	89.22	0.00	0.00	0.00	78.43	0.00	0.00	0.00	0.00	0.00	0.00	0.00	0.00	0.00	0.00
S7	530	5	99.81	0.00	97.36	93.96	86.42	0.00	90.00	0.00	0.00	0.00	0.00	0.00	0.00	0.00	0.00	0.00	0.00	0.00	0.00
S8	182	4	98.35	0.00	0.00	0.00	0.00	0.00	80.22	0.00	0.00	0.00	64.29	0.00	0.00	76.92	0.00	0.00	0.00	0.00	0.00
S9	120	3	100.00	0.00	0.00	0.00	94.17	0.00	0.00	0.00	95.00	0.00	0.00	0.00	0.00	0.00	0.00	0.00	0.00	0.00	0.00
S10	516	3	39.15	0.00	0.00	31.40	0.00	0.00	0.00	12.40	0.00	0.00	0.00	0.00	0.00	0.00	0.00	0.00	0.00	0.00	0.00
S11	709	2	34.84	0.00	0.00	11.99	0.00	0.00	0.00	0.00	0.00	0.00	0.00	0.00	0.00	0.00	0.00	0.00	0.00	0.00	0.00

Table S2.

Percentage of Cases with Teacher-reported Suicidal Thoughts and Behavior by Wave (Coded as Years from Baseline)

Study	*n*	Waves	Wave (coded as years from baseline)																	
			0	0.25	0.5	0.75	1	1.25	1.5	1.75	2	2.5	2.75	3	3.5	3.75	4.75	5	6	7
S1	241	6	96.27	0.00	0.00	94.19	0.00	0.00	0.00	80.91	0.00	0.00	72.20	0.00	0.00	65.98	18.67	0.00	0.00	0.00
S2	97	3	93.81	0.00	0.00	97.94	0.00	0.00	0.00	78.35	0.00	0.00	0.00	0.00	0.00	0.00	0.00	0.00	0.00	0.00
S3	245	0	0.00	0.00	0.00	0.00	0.00	0.00	0.00	0.00	0.00	0.00	0.00	0.00	0.00	0.00	0.00	0.00	0.00	0.00
S4	80	3	100.00	95.00	0.00	0.00	0.00	73.75	0.00	0.00	0.00	0.00	0.00	0.00	0.00	0.00	0.00	0.00	0.00	0.00
S5	360	7	98.33	0.00	0.00	0.00	60.00	0.00	0.00	0.00	78.89	0.00	0.00	47.78	0.00	0.00	0.00	22.50	28.06	30.28
S6	102	3	100.00	0.00	0.00	97.06	0.00	0.00	0.00	78.43	0.00	0.00	0.00	0.00	0.00	0.00	0.00	0.00	0.00	0.00
S7	530	5	98.11	0.00	93.96	87.36	79.43	0.00	78.11	0.00	0.00	0.00	0.00	0.00	0.00	0.00	0.00	0.00	0.00	0.00
S8	182	4	98.90	0.00	0.00	0.00	0.00	0.00	89.01	0.00	0.00	76.37	0.00	0.00	73.63	0.00	0.00	0.00	0.00	0.00
S9	120	3	100.00	0.00	0.00	96.67	0.00	0.00	90.83	0.00	0.00	0.00	0.00	0.00	0.00	0.00	0.00	0.00	0.00	0.00
S10	516	3	75.00	0.00	0.00	78.49	0.00	0.00	0.00	46.90	0.00	0.00	0.00	0.00	0.00	0.00	0.00	0.00	0.00	0.00
S11	709	3	82.09	0.00	0.00	77.15	0.00	0.00	0.00	48.94	0.00	0.00	0.00	0.00	0.00	0.00	0.00	0.00	0.00	0.00
